# Impact of a Vibrotactile Belt on Emotionally Challenging Everyday Situations of the Blind

**DOI:** 10.3390/s21217384

**Published:** 2021-11-06

**Authors:** Charlotte Brandebusemeyer, Anna Ricarda Luther, Sabine U. König, Peter König, Silke M. Kärcher

**Affiliations:** 1 Institute of Cognitive Science, University of Osnabrück, 49069 Osnabrück, Germany; cbrandebusem@uni-osnabrueck.de (C.B.); anluther@uni-osnabrueck.de (A.R.L.); sabkoeni@uos.de (S.U.K.); silke.kaercher@uni-osnabrueck.de (S.M.K.); 2 Department of Neurophysiology, University Medical Centre Hamburg-Eppendorf, 20251 Hamburg, Germany; 3 FeelSpace GmbH, 49069 Osnabrück, Germany

**Keywords:** blindness, assistive technology, vibrotactile belt, safety, orientation, navigation

## Abstract

Spatial orientation and navigation depend primarily on vision. Blind people lack this critical source of information. To facilitate wayfinding and to increase the feeling of safety for these people, the “feelSpace belt” was developed. The belt signals magnetic north as a fixed reference frame via vibrotactile stimulation. This study investigates the effect of the belt on typical orientation and navigation tasks and evaluates the emotional impact. Eleven blind subjects wore the belt daily for seven weeks. Before, during and after the study period, they filled in questionnaires to document their experiences. A small sub-group of the subjects took part in behavioural experiments before and after four weeks of training, i.e., a straight-line walking task to evaluate the belt’s effect on keeping a straight heading, an angular rotation task to examine effects on egocentric orientation, and a triangle completion navigation task to test the ability to take shortcuts. The belt reduced subjective discomfort and increased confidence during navigation. Additionally, the participants felt safer wearing the belt in various outdoor situations. Furthermore, the behavioural tasks point towards an intuitive comprehension of the belt. Altogether, the blind participants benefited from the vibrotactile belt as an assistive technology in challenging everyday situations.

## 1. Introduction

Wayfinding is a highly complex and important activity in everyday life. Success depends on a number of factors, e.g., on a person’s spatio-cognitive abilities, i.e., understanding space and manipulating it mentally [1]. This is especially challenging for those with visual impairments, as they cannot rely on the main source of spatial layout information: vision. According to the WHO, about 30 million visually impaired and blind people live in the EU [2]. Navigation skills are of great importance for partaking in society, e.g., participating in education, the labour market and civic life. Supporting visually impaired people in their ability to find their way in everyday life is therefore vital.

Reaching a destination in outdoor navigation is a time and effort consuming task, as it requires the ability to orientate as well as to navigate. Orientation has been defined as a cognitive process in which all sensory information is used to compute one’s own position relative to other objects in the environment [3]. Blind and visually impaired people cannot rely on vision and must therefore solely rely on touch, hearing and, occasionally, smell [4] which is a severe handicap in orientation tasks. Navigation is associated with directed movement in which one’s position in the environment has to be located with respect to a destination, i.e., moving towards a certain goal. Here, relevant information might be retrieved from memory or deduced by cognitive processes, e.g., through path integration during self-motion [5,6,7]. Without visual cues to correct the direction in which one is heading, small errors in path integration accumulate quickly. Therefore, the lack of visual information weighs heavily in such tasks. Thus, blind and visually impaired people lack crucial information for several components of wayfinding tasks.

Besides these concurrent navigation and orientation challenges for blind people, en route, obstacles—stationary and non-stationary—must be avoided and bypassed. One well-known problem in this context is the detection of and correction for involuntary body turns as well as estimating the degree of intentional body turns, e.g., after walking around an obstacle or before making a turn [8]. There is a variety of aiding devices for the visually impaired, most notably the white cane (e.g., [9,10]), navigation applications for the smartphone (e.g., [11,12]) and auditory compasses [13,14,15]. Further developments of the white cane enable obstacle detection also from a greater distance [16,17,18]. In science, the field of sensory substitution takes this a step further by trying to substitute visual information in its entirety through other senses and thus enabling blind people to have a kind of visual experience (e.g., [19,20,21,22,23,24]). Through active handling and training with the devices, astonishing visual experiences of blind people like object recognition and depth perception (e.g., [19]) were reported. In addition, changes in brain activity due to sensory substitution could be found [25]. However, sensory substitution devices are not widely used by blind people in everyday life [26,27], as they require long training periods and often demand too much attention. For all aiding devices in this context, it is of importance that they neither block hands nor ears since these are the main channels for information gathering for the visually impaired and blind people [28]. Taken together, there are several aiding devices for blind people and for the visually impaired to overcome different aspects of difficulties in wayfinding and orientation in everyday life.

The high amount of attention that is needed to process the independent factors of assistive devices in parallel makes orientation and navigation outdoors for blind and visually impaired people difficult tasks [4]. At each decision point of a route, the blind traveller has to make correct estimations and decisions about their heading to successfully reach a destination, whereas the probability of an erroneous estimation rises with the number of decisions points en route [29]. As blind participants cannot visually adjust those imprecisions e.g., using landmarks, the associated uncertainty can be overwhelming, and often leads to stress, discomfort or even anxiety for the blind [1,29,30,31]. These negative emotions occur when environmental demands rise beyond the available coping resources. They often limit partaking in everyday life for the visually impaired e.g., leisure activities, entertainment, or visiting friends or family due to the stress associated with getting there [31]. To adapt to wayfinding without visual cues, Orientation and Mobility training is offered for the visually impaired to practise coping strategies. It has been shown that Orientation and Mobility training has an impact on stress levels concerning the usage of public transportation, but not directly on wayfinding when walking [31]. During everyday wayfinding, there is no immediate feedback on the performance, e.g., for turning a certain degree or following a direction. Thus, the blind person might objectively be well adapted and yet feel subjective high levels of stress. Immediate feedback on performance underways, such as whether one is keeping to the direction in which one is heading, turning degrees and directions, potentially reduces stress and discomfort and decreases the described avoidance behaviour. All in all, direct feedback on orientation performance could thus have a strong impact not only on navigation and orientation success but also reduce the stress level for outdoor activities and thus increase the quality of life.

One way to overcome problems during outdoor navigation, as outlined above, is to provide the blind person with a constant reference frame, e.g., through signalling a fixpoint for body axis alignment and information on the direction of movement. A potential candidate to fill this gap is the feelSpace belt [32,33,34]. The feelSpace belt was developed as a sensory augmentation device that provides a continuous vibrotactile signal about the direction of the magnetic north around the waistline. Previous research with the feelSpace belt and sighted participants revealed that the subjects developed a new sense of space perception, especially concerning egocentric and allocentric spatial relations [33,34]. Sighted participants reported a strong feeling of security of “never getting lost again” while they were wearing the belt [33,34]. The influence of the feelSpace belt’s information was also tested with a late blind participant [35] and a congenitally blind participant [36]. Both participants showed improved mobility and orientation abilities. They also reported a feeling of increased security in outdoor navigation when taking a shortcut across an open space with the constantly available north signal [35]. Thus, the feelSpace belt provides a continuous reference towards the north that blind people can use as an update on their own orientation in space while moving, resulting in a feeling of security in outdoor navigation and wayfinding.

In the present paper, we want to evaluate the impact of the feelSpace belt on the stress that is commonly associated with outdoor navigation and on the navigation and orientation performance of blind participants. To this end, we equipped blind participants with the feelSpace belt for seven weeks and instructed them to use the belt as much as possible in outdoor activities in their daily life. We evaluated their subjective experiences with the belt, especially their sense of security and discomfort in everyday situations of traffic, with previously tested questionnaires [35]. We additionally investigated the performance of a subgroup of participants in behavioural tasks (straight-line-walking, angular rotation and a triangle completion task) to test whether the belt influenced their orientation and navigation abilities before and four weeks into the study. We hypothesize that the continuous, reliable signal of the feelSpace belt on cardinal north as a tangible fixpoint can be intuitively understood and used to adjust one’s heading, improves the feeling of security in everyday outdoor tasks and also improves aspects of spatial orientation and navigation for the blind participants.

In the present paper we examined three aspects:

1. the emotional impact of the belt during everyday outdoor navigation
2. the intuitiveness of the belt use
3. the impact of the feelSpace belt on navigation and orientation performances

## 2. Materials and Methods

### 2.1. Participants

Eleven subjects (four female) with a mean age of 54.36 years (SD = 11.96, range 34–73) took part in this study. Five out of the eleven subjects were congenitally blind while the other six subjects were late blind. All eleven subjects recorded their experience in questionnaires. Due to the limitations imposed by the Covid-19 pandemic, we could only perform the behavioural tests with five subjects (one female, one congenitally blind). All participants were active and mobile people that had received Orientation and Mobility training prior to this study (range 25–300 h) and had been blind for several years (mean 39.5, SD = 15.5, range 20–60 years). The high amount of mobility training hours of some participants is due to them having moved and therefore having taken additional training hours to adapt to the new environment. They are therefore not to be taken as an indicator for the participants’ individual orientation abilities. All participants knew cardinal directions, angles and degrees, which were essential prerequisites to be able to interpret and understand the vibration signal of the belt towards the north. All use the white cane as an aid in outdoor activities and use traffic noise for guidance. They were familiar with urban challenging situations such as crossing large junctions, roads, open spaces or walking along train platforms and all but one rated themselves as being able to face these challenges by themselves without another person’s help. The participants rated their sense of orientation from quite good to very good before the study. All participants gave written informed consent to participate in this study and the Ethics Committee of the University of Osnabrück gave approval following the Institutional and National Research Committee’s ethical standards (#4/2019 and #26/2020).

### 2.2. Technical Setup

**Figure 1:**
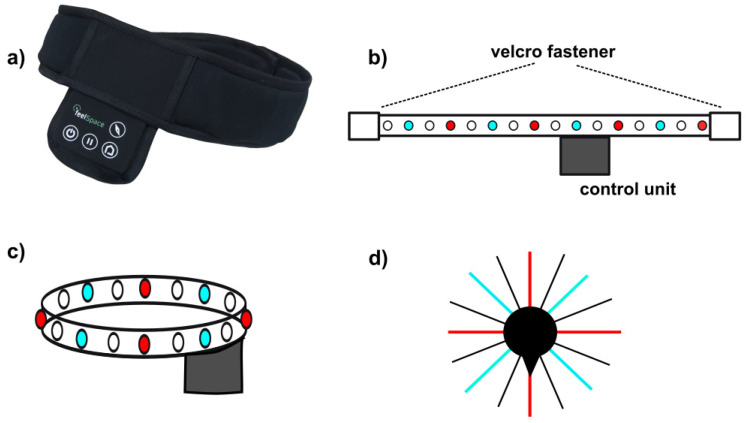
The feelSpace belt [37]: (**a**) picture of the belt, (**b**) layout when stretched open, (**c**) vibration motor layout around the waist, vibration motors on 90° in red, on 45° in light blue, control unit marked in grey, (**d**) vibration layout around the body axis, walking direction is marked with the black triangle.

The feelSpace belt provides directional information of the magnetic north via vibrotactile stimulation around the waist (Figure 1) and is distributed by the company feelSpace (feelSpace, Osnabrück, Lower Saxony, Germany). The vibrotactile information of the investigated belt is given by sixteen vibromotors that are evenly spaced around the waist covering 360°. One vibromotor thus covers 22.5° of the circumference (Figure 1). An integrated compass senses the direction of the magnetic north and transfers this information to the northernmost vibromotor. Thus, only the single northernmost module vibrates at a time. To ensure that all vibrotactile motors were felt also on the back, participants were asked for their feedback on this point at the beginning of the study and the vibration strength was adjusted accordingly. Furthermore, participants could adjust the strength of the vibration to their needs themselves. In previous research with the feelSpace belt, the feedback of participants showed that they did not have problems to distinguish between neighbouring vibromotors even with 30 motors equally spaced in the belt. The current feelSpace belt is a development of the original design that included thirteen vibromotors [32]. To ensure usability during everyday life indoor and outdoor, the belt uses a long-lasting battery of at least 8 h and up to 20 h, is weatherproof and is made of skin-friendly fabric (for further technical details see [35]).

The current belt has two main modes of operation: The standard is the compass mode, in which the direction of the magnetic north is indicated via a continuous vibration of the northernmost motor. When the user turns his/ her body axis, this signal “turns” accordingly around the body to accommodate the turn. The second mode is the road-crossing mode. When switched on, the road-crossing mode transmits pulsing vibrotactile stimulation precisely at the navel as the centre of the belly. The vibrotactile signal “points” in a straight line forward and can be used to cross a road or a large open space. If the blind person diverges from the straight line, the vibration signal diverges to the left or the right, indicating the direction and extent of the deviation.

The belt can be connected to the feelSpace smartphone application to use the belt to receive directions for navigation. This function, however, is not in the focus of this present study. All in all, the feelSpace belt enables the users to follow their gut feeling in the literal sense.

In order to measure the turning angles and the turning direction of the participants, the application “Compass Experiment” was developed. During the angular rotation task, the application on the smartphone gave instructions to the participant in which direction to turn. The participant held the smartphone and pressed the screen to receive the next instruction. The feelSpace belt could be connected to the application during the experimental tasks. The application then recorded the time needed for turning, the present orientation, the difference in turning degree to the start orientation and the orientation at the end of the last turn, the turning degree and the turning direction (left or right). With this information, turning errors could be calculated. This application also ensured that no interaction between participant and experimenter was present. A subconscious impact of the experimenter on the performance of the participants can therefore be obviated.

### 2.3. Study Procedure

**Figure 2:**
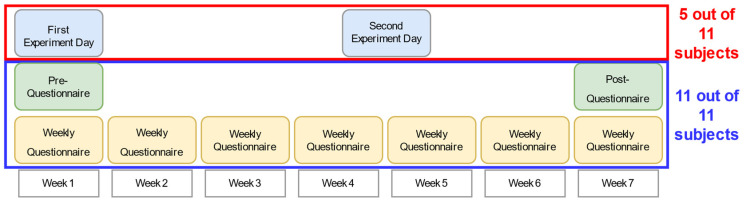
The overall schedule and structure of the study: The weekly questionnaires were filled in by all eleven subjects and five of the eleven subjects additionally took part in behavioural experiments at the beginning and after four to five weeks of the study.

The participants were instructed to fill in a pre-questionnaire, concerned with the medical history, orientation strategies and the self-assessment of emotions in various situations of traffic. During the seven weeks (Figure 2), participants were instructed to wear the belt in every waking hour and wore it in known and unknown environments. Each week they filled in a questionnaire with questions regarding different situations in which they had to orientate and navigate and their feeling of security in various situations when equipped with the belt. These questions remained the same every week to be able to detect changes in the participants’ evaluations over the weeks. At the end of this training period, the participants filled in a final post-questionnaire. The questionnaires were all designed in SurveyMonkey, an online platform for designing barrier-free questionnaires.

Five subjects additionally took part in behavioural experiments (Figure 2) at the University of Osnabrück. The participants were informed about the procedure of the study, gave their consent to participate and filled in the pre-questionnaire with one of the experimenters. First, the subjects completed a straight-line walking task, then an angular rotation task and finally did a combination of a triangle completion and pointing task. Every task was performed under a variety of conditions: with the belt in compass mode or without the belt and with or without distraction tasks. The straight-line walking task was additionally performed with the belt in road-crossing mode. After each condition the subjects were asked about their sense of security. The three experiments were performed again on the second experiment day after the participants had trained for four to five weeks with the belt.

Due to the COVID-19 pandemic, specific safety measures had to be taken for the experiment days in accordance with the Corona policy in Germany and Lower Saxony of 22 March 2020. The experimenters, as well as the participants, wore masks during the whole experiment and kept a distance of at least 1.5 m. Furthermore, each participant had contact to only one of the experimenters during the experiment to further diminish the risk of infection. Hands and utilities were disinfected before and after every participant and the experimenters wore sanitary gloves during the experiments.

### 2.4. Study Design

#### 2.4.1. Questionnaires

The questionnaires consisted of multiple-choice questions, matrix questions with quantitative Likert scales and open-ended qualitative questions. A pre-questionnaire administered before the study served as a source of information about the participants that took part in the study. The length and degree of blindness, the orientational aids (such as a cane or dog) and orientational strategies (e.g., listening to traffic or using a smartphone) of the subjects and their emotions when encountering various situations of traffic were addressed. The weekly questionnaire was mainly based on the one employed by [35]. The questions can be subdivided into four categories: the perception of the belt during the week, questions concerning emotions and motivation, changes in orientation abilities and the use of attentional resources. Finally, a post-questionnaire was conducted in which the participants could give their final feedback on the feelSpace belt and on the study design and procedure. In this paper, the focus is laid on the questions concerning confidence and discomfort in various situations of traffic, the sense of security and the improvement of orientational abilities. Further, we investigated whether the amount of time spent in foreign surroundings and familiar surroundings correlates with the perceived benefit of the belt.

Since discomfort is widely reported in situations of traffic by the blind, we invested the development of discomfort and confidence when crossing a road, crossing a junction with multiple lanes, traversing an open space and walking along a train platform with Likert scaled statements, i.e., “*I feel discomfort when I […].”* and *“I feel confident when I […]”*.

The sense of security is investigated through the statements *“I feel secure in foreign surroundings.”* from the pre-questionnaire and *“With belt I felt more secure in foreign surroundings than without.”* from the weekly questionnaire. The perceived improvement of orientational abilities was addressed with questions both about specific situations requiring different orientational abilities and questions about a more general sense of orientational abilities. Questions on specific situations encompass the following questions: *“With belt it is easier to notice that I walk around a long bend.”*, *“With belt I am always conscious of where I am in relation to my home.”*, “*With belt it is easier to estimate the position of streets/places to each other.”* and *“With belt it is easier to find my way in foreign surroundings.”.* A potential improvement of a more general sense of orientation is addressed through the following questions: *“Since I wear the belt, I am more aware of cardinal directions.”*, *“My spatial orientation has subjectively improved, since wearing the belt.”* and *“When I take the belt off, my spatial orientation worsens.”*. In the post-questionnaire the participants could give their final feedback on the feelSpace belt and on the study design and procedure. Finally, the amount of time spent in familiar and unfamiliar surroundings is collected through the weekly questionnaires and the evaluation of the benefit of the belt in the respective situation is evaluated through the statements *“The belt helped especially in (un)familiar surroundings.”*.

All questionnaires and their mode of transmission were additionally approved by a rehab teacher working at a school for blind people.

#### 2.4.2. Behavioural Experiments

The three behavioural experiments took place on an open space outdoors. The surrounding was not familiar to the subjects. After every experiment, we asked the participants which orientation strategies he/she employed for the specific task. This way we checked that they used egocentric strategies whilst ignoring surrounding potential cues. Furthermore, neither the subjective reports nor our data analysis indicate that learning the specific tests influence the experimental results in a relevant way.

#### 2.4.3. Straight-Line-Walking Task

During the straight-line-walking task participants try to keep to the direction in which they are heading. The task aims at mimicking the crossing of a large street or an open space. Keeping a straight line is challenging for the blind as they cannot refer to visual landmarks to steady their heading.

**Figure 3:**
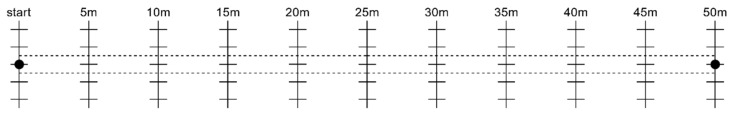
The general set-up of the straight-line walking task is depicted. The field was 50 m long and 6 m wide. The dotted lines indicate the ideal walking corridor. Deviations from this walking lane were documented every 5 m. If the subjects diverged more than 3 m from the ideal straight heading, the trial was aborted.

For the straight-line walking task, a large grid was drawn on the ground outdoors (Figure 3). The subject was initially aligned towards the goal point by the experimenters. The subjects were instructed to keep to the initial direction until they reached the goal point. Two practice trials, one with the activated belt and one without the activated belt, were conducted to get the participants acquainted with the procedure of the task. Every participant then performed 12 trials, two trials per condition. The six conditions were: Without belt (with and without distraction), with belt in the compass mode (with and without distraction) and with the belt in the road-crossing mode (with and without distraction). As a distraction, the participants performed a mental calculation task by subtracting 3 repeatedly from 1000. The participants were instructed to calculate as fast and as precisely as possible and to say each result out loud. The trials with a distraction task were always performed first to reduce the probability of a training effect. To ensure a natural setup, the participants walked with their own white cane since this is an aid they use and rely on outdoors. To indicate the direction in which to head and give the start signal, one of the experiment conductors gave a “go”-signal from the finishing point. One experimenter followed the subject from the starting point onward, stopping the subject if the limits of the experimental field were reached, and documenting the walking path of the subject. During the trials, the time was measured from the beginning to the end of the field and each trial was video recorded. The performance of the participants was analysed according to their walking distance.

#### 2.4.4. Angular Rotation Task

The angular rotation task was designed as a direct measure for turning estimations and as an indirect measure for orientation abilities of the participants. Specifically, the effect of the belt on egocentric orientation, meaning orientation based solely on idiothetic cues, are examined.

**Figure 4:**
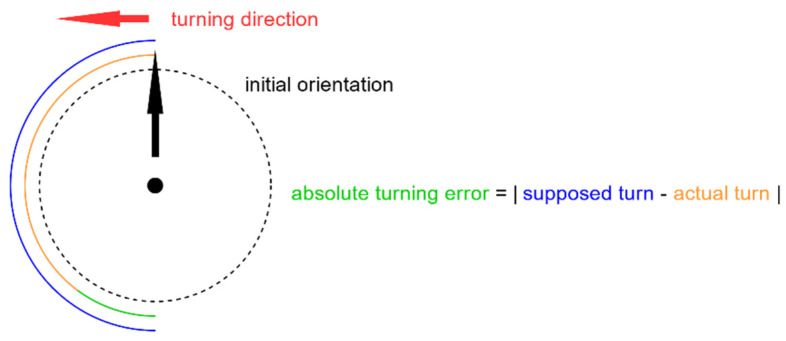
During the angular rotation task, the participant stood in a random direction. Via the Compass Experiment application, the participant was told in which direction and how far to turn from his initial position—either 90°, 180° or 360°. The absolute turning error was calculated by taking the absolute value of the difference between actual turning angle and the instructed turning angle.

The participant’s belt was connected to the “Compass Experiment” application. The participant held the smartphone in his/her hand. Via the application, he/she was instructed to either do a quarter turn (90°), half a turn (180°) or a whole turn (360°) either to the left or the right. The subject then tried to rotate in an as exact angle as possible and touched the screen of the smartphone to indicate the final direction. All in all, the subject did 12 turns without the belt and 12 turns with the belt in compass mode (Figure 4). The order of the conditions without belt and with belt was randomized across participants but remained the same for the first and the second day. The initial orientation of the participant was also random. The reason for the application giving the instructions and not one of the experiment instructors was, that this way, there was no external reference point that the participant could use as an auditory cue during the task. Four practice turns—two without the belt signal and two with the belt signal—were performed beforehand to introduce the subjects to the application and the type of instructions. The absolute turning error was calculated to measure the participants’ performance.

#### 2.4.5. Triangle Completion Task

With a triangle completion task, the impact of the feelSpace belt on the participants’ spatial understanding and navigation abilities was examined by mimicking a person taking a shortcut.

**Figure 5:**
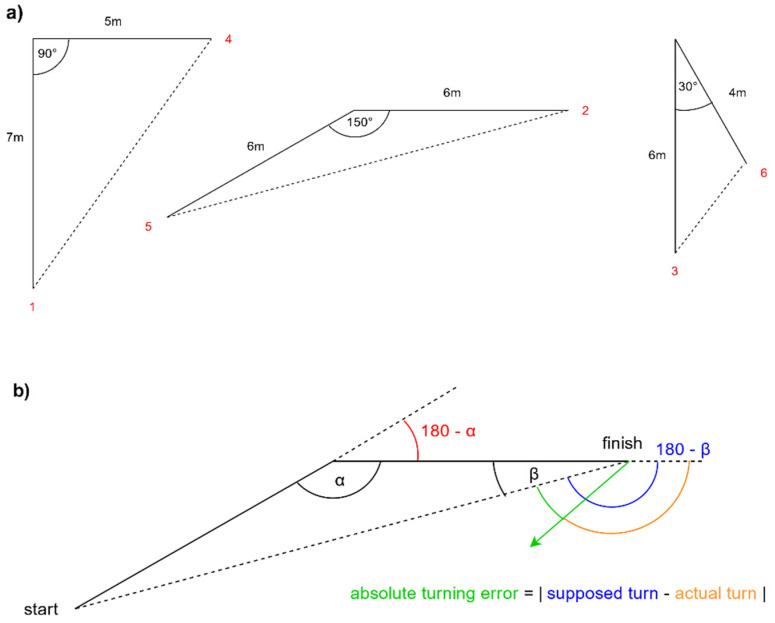
The participants were led along the sides of the three triangles with inner angles of 90° (right-angled triangle), 150° (isosceles triangle) and 30° in the order of the red numbers (**a**). The turns which the participants had to perform are depicted in (**b**). At the finishing point, the participants had to turn back to the starting point. The absolute difference between the actual turning angle and the instructed turning angle results in the absolute turning error (**b**).

The experimenter led the subjects with a stick along two edges of a triangle, which was drawn on the ground (Figure 5). The end of the second edge of the triangle was signalled by the experimenter taking down the stick. Upon this signal, the participant turned back towards the estimated starting point. Whereas in former studies, participants had to walk back to the point where they estimated the starting point, in our study we only asked them to turn into the direction they thought the starting point was since only their direction estimation was of interest. The stick was used to exclude the possibility that movements of the experimenter give the participant directional cues [35]. The turning degree of the participant from the endpoint back towards the starting point was recorded with the help of the “Compass Experiment” application and the belt connected to it.

The tests employed three triangles with varying inner angles (90°, 150° and 30°, see Figure 5a). The subjects walked the triangles in both directions, resulting in six trials with the inner angles being either on the left- or the right-hand side of the participant. The executed conditions were with belt with distraction, with belt without distraction, without belt with distraction and without belt without distraction. The distraction task consisted of the same calculation task as in the straight-line walking task and was always performed first to avoid a training effect. The participants were instructed to calculate as fast and as precisely as possible and to say the result out loud. Again, the conditions were randomized between subjects but identical within subjects on both experiment days. The absolute turning error (Figure 5b) was used as the error measure.

### 2.5. Data Analysis

Quantitative data from the Likert scaled questions in the questionnaires was analysed over all eleven participants. Descriptive analyses, as well as statistical measures were performed in MATLAB R2020a and IBM SPSS Statistics 26. For group comparisons, the Mann–Whitney U-test was used and for paired samples, the Wilcoxon test was applied. Missing data was filled with values of the previous week; thus, no change was assumed when a value was missing. To test for correlations, Kendall’s T_b_ was applied.

The open-ended questions served the purpose of gathering the participants’ experiences, problems and wishes and served as a source of subjective impressions. All quotes from the open-ended questions as given in the results section have been translated from German to English. The original quotes in German can be provided on inquiry. It is important to keep in mind that blind people are a very heterogeneous group in which each person has individual strategies to cope with his/her impediment.

The five participants who took part in the behavioural experiments could be subdivided into four late blind participants and one congenitally blind participant. The late blind participants will be analysed as a group while the results of the congenitally blind subject will not be presented in this paper. This is due to the very different situation for the congenitally blind subject in the behavioural test, because of him never having seen and therefore never having used visual cues to base his orientation strategies on in contrast to the late blind participants. Descriptive statistics were performed in Excel 2019. To test for statistical significance between conditions and experiment days, repeated measure analysis of variance (RM-ANOVA) was calculated with IBM SPSS Statistics 26.

## 3. Results

### 3.1. Subjective Data

Eleven blind participants gave their subjective reports during seven weeks of training with the feelSpace belt. In the following, the focus of analysis will be laid on the emotional impact of the belt on the participants, especially on their emotions during critical traffic situations and their sense of security, and on their subjective evaluation of changes in their orientation abilities.

#### 3.1.1. Situational Emotions

**Figure 6:**
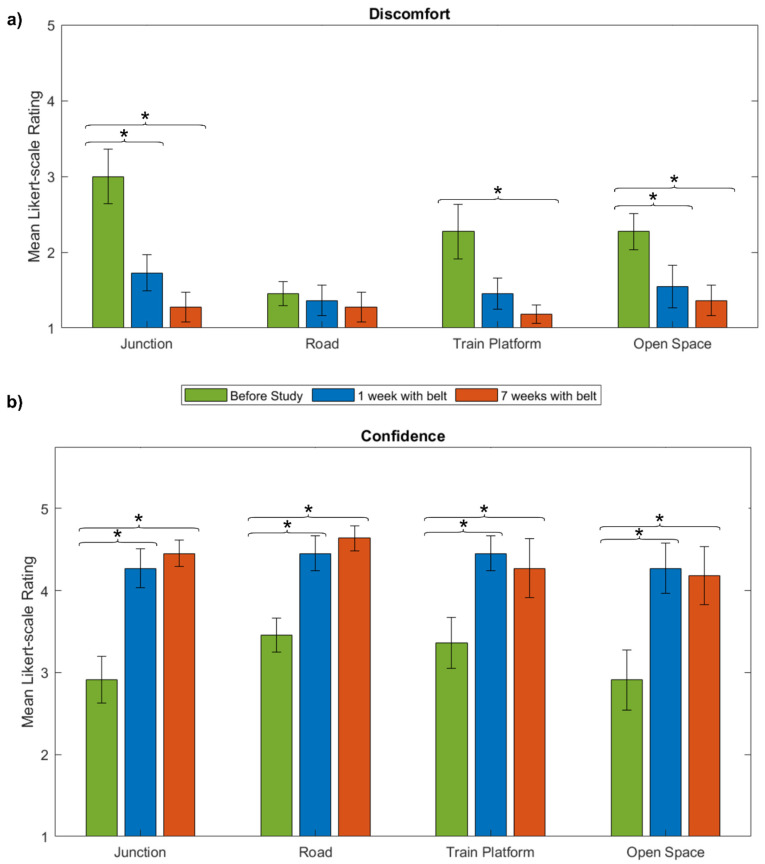
(**a**) The mean Likert scale ratings with the standard error of the mean (SEM) of the self-assessment to what degree the participants felt discomfort when crossing a junction with multiple lanes, crossing a road, walking along a train platform and crossing an open space without any external directional cues before the study (green), after one week with belt (blue) and after seven weeks with the belt (orange) are displayed. (**b**) The mean Likert scale ratings with the SEM of the self-assessment to what degree they felt confident when in the respective situation before the study (green), after one week with belt (blue) and after seven weeks with the belt (orange). The asterisk indicates a significant difference (*p* < 0.05).

Visually impaired extensively report discomfort and a sense of insecurity in everyday traffic situations. Here we study the influence of the belt on discomfort and confidence. Therefore, the participants were asked to rate the degree of discomfort/confidence before the study and then weekly evaluate to what degree they experienced discomfort and confidence in four everyday situations of traffic. These situations included crossing a junction with multiple lanes, crossing a road, walking along a train platform and crossing an open space without any external directional cues while wearing the belt (Figure 6).

Discomfort while crossing a junction with multiple lanes significantly decreased after one week and further decreased after seven weeks of training (*p* = 0.0156, *p* = 0.002, Wilcoxon signed-rank tests, respectively; Figure 6a). Similarly, discomfort in open spaces showed a significant decrease after one and seven weeks of training (*p* = 0.0156, *p* = 0.0176, respectively). Discomfort while walking along a train platform showed a decrease after one week; however, this was not significant, and a further significant drop after seven weeks of training (*p* = 0.938 *p* = 0.0273, respectively). When crossing a road, the ratings were low before and throughout the study.

We observe an increase in confidence ratings in all four situations over the course of the study (Figure 6b). Comparing mean ratings after one week to the level of subjective confidence before to the training revealed a significant difference in all situations (junction: *p* = 0.0313, road: *p* = 0.0469, train platform: *p* = 0.0313, open space: *p* = 0.0313). After seven weeks of training the confidence stayed high and was significantly different from pre-training levels for all situations except for the train platform (junction: *p* = 0.0078, road: *p* < 0.001, train platform: *p* = 0.1875, open space: *p* < 0.001). Hence, the belt gave the participants a feeling of confidence in typical traffic situations after a short training period.

In sum, an immediate emotional improvement became evident both in the decrease of discomfort ratings and the increase of confidence ratings in various everyday situations of traffic. This positive effect either remained stable or became more pronounced throughout the course of the study.

#### 3.1.2. Sense of Security

Orienting and navigating in unknown surroundings are especially challenging tasks for the blind. The pre-questionnaire addressed how secure the participants felt in unknown surroundings before being equipped with the belt. This assessment could then be matched to the corresponding question in the weekly questionnaire allowing to track the development of the sense of security in unknown surroundings. The sense of security significantly increased after one week with the belt (*p* < 0.0001, Wilcoxon signed-rank test). The ratings remained high throughout the study with the mean rating ranging between 3.5 and 3.8. Consequentially, the ratings before the study are significantly lower than after seven weeks with the belt, indicating that the belt helped the participants to feel more secure in unknown surroundings.

#### 3.1.3. Changes of Orientation

To assess the subjectively perceived improvement of orientation during belt training, we evaluated the corresponding questions of the weekly questionnaires.

**Figure 7:**
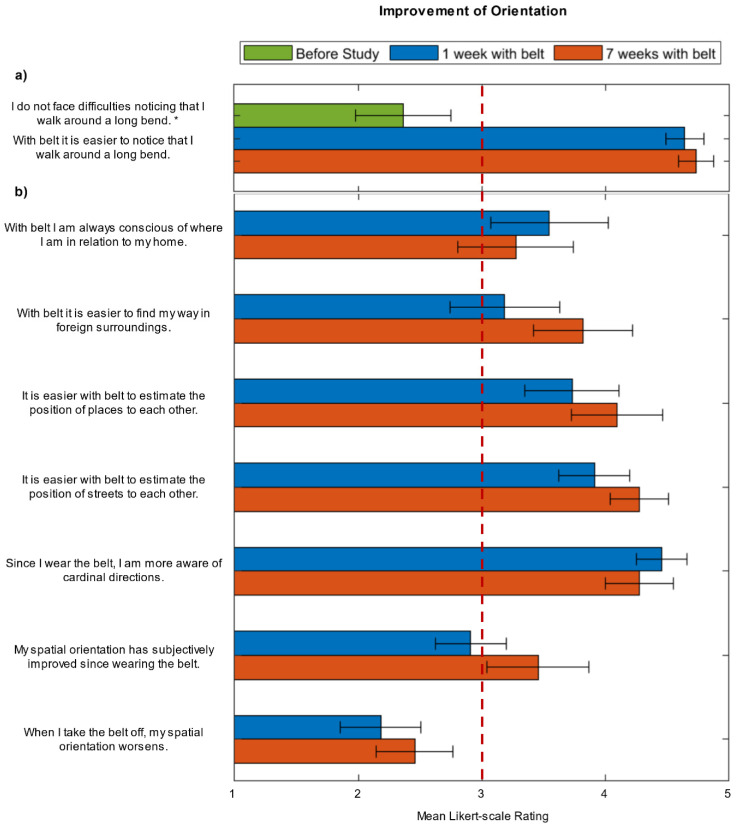
(**a**) The mean Likert scale ratings with the SEM express the participants’ own evaluations of the difficulty to detect a long bend before having been in contact with the belt i.e., before the study (green) and the facilitation experienced in the respective task through the belt after one week with belt (blue) and after seven weeks of wearing the belt (red) i.e., after the study. (**b**) The mean Likert-Scale ratings with the SEM of the self-assessment of improvement in orientation after one week of training (blue), and after seven weeks of training (red) are depicted. * Inverted values of the original question “I face difficulties noticing that I walk around a long bend” are depicted.

One question concerning the ability to detect a long bend was assessed in the pre-questionnaire as well as in the weekly questionnaire. We compared evaluations of the participants before training with the belt, after one week of training and after seven weeks of training (Figure 7a). Before the training subjects strongly denied the statement that they do not have any difficulties noticing a long bend, i.e., confirming that long bends are difficult to navigate. After training with the belt, they strongly supported the statement that the belt makes it easier to notice such a bend. Thus, the belt is perceived to be beneficial when detecting a long bend from the first week on and continuously so throughout the study. The seven remaining questions directly refer to the belt and therefore do not have a matching counterpart in the pre-questionnaire. Thus, we compare the ratings after one and seven weeks of training with the belt (Figure 7b). Overall high ratings are obtained in six out of seven questions both after one week with the belt and after seven weeks with the belt. Of the questions addressing potential improvements due to the belt all but one are rated above average (Figure 7). Furthermore, no significant changes are obtained throughout the study employing the Friedman test or when comparing week one with week seven employing the Wilcoxon signed-rank test (*p* > 0.05). Nominally, all ratings are higher in the following weeks than after the first week.

Summing up, the self-assessed improvement in various aspects of orientation was high after one week with the belt and remained high throughout the study.

#### 3.1.4. Time Spent in Familiar and Unfamiliar Surroundings

During the seven weeks, the eleven participants wore the belt in familiar as well as in unfamiliar surroundings. Most participants remained mostly in familiar environments (mean: 89.43 h during the seven weeks), but all participants spent at least some time also in unfamiliar environments (mean: 29.77 h during the seven weeks). The absolute wearing time of the belt in known and unknown surroundings did not coincide with the participants finding the belt most helpful in the respective surroundings (known surrounding: T_b_ = 0.342, *p* = 0.167; unknown surrounding: T_b_ = 0.181; *p* = 0.465). No correlations between the amount of time spent in known and unknown surroundings and questions of the weekly questionnaires were observed.

### 3.2. The Participants’ Experiences during the Training Period

To grasp the impact of the belt on the participants more deeply, some impressions and experiences of the participants during the seven weeks of wearing the belt are presented in the following. Some exemplary quotes from different participants are selected to illustrate the subjective impact.

The participants described the impact of the belt in different situations involving traffic and the feeling of safety that the belt conveys. Participant 7 (P7) stated: “*The knowledge of always being able to get fast and reliable feedback on my orientation conveys to me a feeling of safety […].”* P4 stated in the third week that “*in all critical situations in traffic such as road and rail crossings as well as crossing open squares, the belt helped me very much*.” “*With no perception of light, the belt is indispensable to monitor whether one is keeping to the right direction. When walking with the cane, the belt gives, to a very large extent, an additional feeling of safety in known and unknown environments—especially in traffic*”, P4 further concluded at the end of the study. Through this additional feeling of safety one participant (P1) gained more independence due to wearing the belt. In the fifth week, he learned a new way to walk to work via a large complicated triangular crossroad and through the woods; he said that he tried it only because of his feeling of safety with the belt: “*I crossed a confusing triangular crossroad several times and learned, with the help of the direction signal* [of the belt]*, to safely cross it.*” (P1). This further illustrates the increased courage to try new things with the tactile compass belt.

However, there were also limitations mentioned. P3 and P10 pointed out that one should not rely absolutely on the belt’s signal. At crossroads with pavements not aligned to the road or pedestrian crossing at an angle from the starting position, there is the danger of walking towards the middle of the crossroads if one relies only on the belt’s signal and does not know the crossroads, P10 pointed out. P3 stated that he would not rely on the belt when walking along a train platform without guiding lines: “*Both the signal of the belt and its perception are not exact enough to keep a constant distance to the edge of the platform*. […]” In the process of orientating and navigating in urban situations as well as in the countryside, the belt helped to keep track of the direction in which one is heading. In conclusion, it is important to bear in mind that the belt is only an aid which offers additional information but can replace neither mobility training nor the cane.

Turning to the participants’ perception of the signal of the belt, P4 directly transferred the information of the belt into spatial information. “*One directly transfers the signal into direction*”, P4 stated in the first week. Concerning the impact on walking straight: *“The registering of bends and turns* etc. *helped to better comprehend and thus to internalize a path one walked along*”, P2 said when asked for situations in which the belt was of help.

The participants further commented on their orientation abilities: The belt facilitated orientation from an egocentric perspective because “[…] *I was at every point in time aware of either the planned or the actual direction in which I was moving*”, according to P7. P8 found two aspects that influenced her orientation strategies. Firstly, she could concentrate better on other things when orientating (counting the number of crossroads and crossings, finding and memorising specific points along the route) and secondly, she could use different paths which she had avoided previously because there were no landmarks to use for orientation. Concerning her navigation abilities, P3 commented that her “*perception of the network of roads had improved and clarified, also in an environment that has been familiar to [her] for years.*”. This perception supports the general impression of the participants that with the belt it is easier to estimate the position of roads to each other. Three participants made comments on how the belt positively affected their inner map. P7 for example stated: “*The knowledge of always being able to get a fast and reliable feedback on my orientation […] synchronises my inner map with reality*.” He further said that his inner map was completed by the belt. In this case, the participant experiences the belt as being helpful for navigational purposes as well as for receiving a feeling of safety whilst navigating.

All in all, these personal experiences highlight both the benefits of wearing the belt as well as an awareness of the limitations of the device.

### 3.3. Behavioural Results

We behaviourally investigated straight-line walking, angular rotation and triangle completion abilities on the first day and after four weeks of belt training. Neither the subjective reports nor our data analysis give an indication that learning the specific tests influence the experimental results in a relevant way.

### 3.4. Behavioural Results of the First Experiment Day

#### 3.4.1. Straight-Line Walking Task

**Figure 8:**
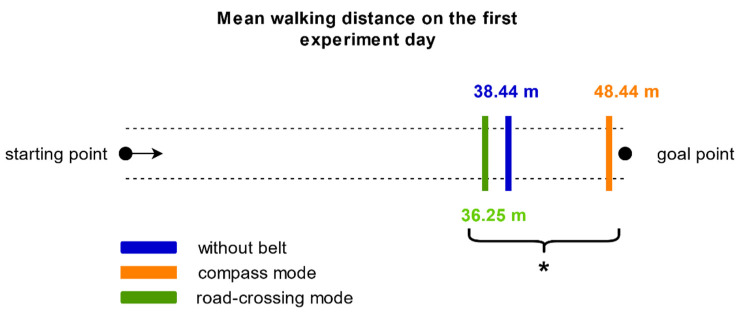
The mean walking distance of the blind participants on the first experiment day for the three belt conditions without belt (blue), with belt in compass mode (orange) and with belt in road-crossing mode (green) is depicted. The asterisk indicates a significant difference (*p* < 0.05).

The straight-line walking task was designed to assess whether the tactile information of the belt helps blind people to keep to the direction in which they are heading and whether performance depends on attentional mechanisms. To evaluate the difference in walking distance, before major deviations from the straight direction, between the six conditions, a 3 × 2 RM-ANOVA with the dependent factors belt condition (without belt/compass mode/road-crossing mode) and distraction condition (with distraction/without distraction) was calculated. The results showed no significant differences between the distraction conditions (*p* = 0.189) but a significant main effect for the belt condition (*p* = 0.047). No significant interaction belt*distraction was found (*p* = 0.264). Since the two-way 3 × 2 RM-ANOVA did not show a significant difference between the distraction conditions nor a significant interaction between belt and distraction, the average of the distraction conditions was taken for further comparisons. Except for one trial, the participants walked the whole distance every time when wearing the belt in compass mode, resulting in an average distance of 48.44 m (Figure 8). The conditions without belt (mean: 38.44 m) and with the belt in road-crossing mode (mean: 36.25 m) were comparable. For post-hoc comparison paired *t*-tests, which compared the belt conditions (without belt/compass mode; without belt/road-crossing mode; compass mode/road-crossing mode), revealed a significant difference between compass mode and road-crossing mode (*p* = 0.041) but no differences in the other two cases (*p* > 0.05). The participants walked straight for a significantly further distance when wearing the belt in compass mode than wearing the belt in road-crossing mode. These results indicate that the compass mode was intuitively accessible and supported straight walking.

#### 3.4.2. Angular Rotation Task Results

**Figure 9:**
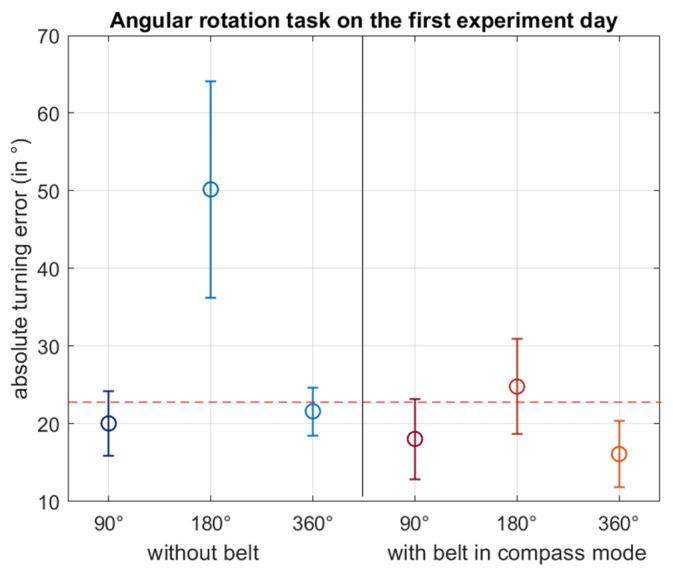
This figure shows the absolute turning errors of the participants during the angular rotation task on the first experiment day. The errors are subdivided into the three turning degrees 90°, 180° and 360°. Turns performed without the belt are displayed in the left half and turns performed with the belt in compass mode are depicted in the right half of the diagram. The circles indicate the mean and the whiskers the SEM. The red dotted line at 22.5° indicates the angular range that the correctly activated single vibromotor covers before the vibrating signal moves to the neighbouring vibromotor.

The angular rotation task was performed to test the direction estimation accuracy and therefore the egocentric body-centred orientation abilities of the participants. No significant differences between left and right turns were found; thus, they will be analysed together. In Figure 9 we see large errors for 180° turns. The mean absolute turning errors of 90° and 360° turns are below 22.5° in both conditions, which indicates that the participants were precise in estimating their egocentric turns already without belt but also with the belt. However, a two-way 3 × 2 RM-ANOVA with the supposed turning degree (90°/180°/360°) and the belt condition (without belt/compass mode) as dependent factors neither revealed a main effect of turning degree or belt conditions nor a significant interaction (all *p*s > 0.05). Thus, on the first experiment day, there is no significant difference in turning accuracy with belt or without and no significant difference between the angular turning degrees.

#### 3.4.3. Triangle Completion Task Results

**Figure 10:**
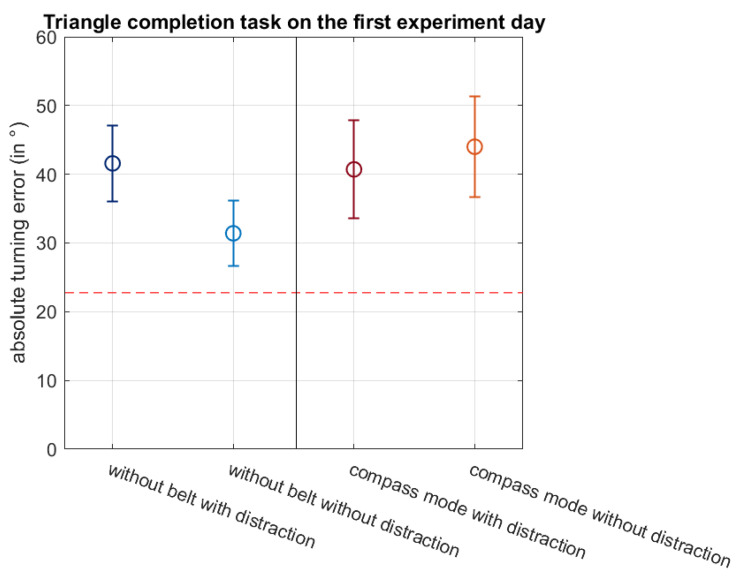
This figure shows the absolute turning errors of the participants during the triangle completion task in the four conditions without belt with distraction, without belt with distraction, with belt with distraction and with belt without distraction on the first experiment day. The circles indicate the mean and the whiskers the SEM. The red dotted line at 22.5° indicates the angular range that the correctly activated single vibromotor covers before the vibrating signal moves to the neighbouring vibromotor.

The triangle completion task addresses the question of whether the belt helps the participants to keep track of their positions whilst moving in an environment, allowing them to take shortcuts. In all four task conditions, the mean absolute errors when turning back to the starting point of the traversed triangle are above 22.5° (Figure 10). Thus, the appropriate vibromotor was mostly not activated when the participants received the vibrating signal via the belt. However, a two-way 2 × 2 RM-ANOVA with the dependent factors belt condition (without belt/compass mode) and distraction condition (with distraction/without distraction) revealed no main effect in the belt condition (*p* = 0.389), no main effect in the distraction condition (*p* = 0.560) and no significant belt*distraction interaction (*p* = 0.233). We conclude that on the first experiment day, the participants’ path integration abilities are equally unprecise without and with the signal of the belt and that the distraction task had no effect on the participants’ performance.

### 3.5. Comparison of First and Second Experiment Day

In the following, the performance of the subjects before training and after four to five weeks of training with the belt is compared. The question to answer is whether the execution of the three experimental tasks differs between the experiment days.

For the straight-line walking task, a three-way 3 × 2 × 2 RM-ANOVA was calculated with the three dependent factors belt (without belt/compass mode/road-crossing mode), distraction (with distraction/without distraction) and day (first experiment day/second experiment day). No significant main effects could be found for the factors day and belt and no significant interactions between any two factors nor between all three factors could be found (*p* > 0.05). However, there was a significant difference between the distraction conditions (*p* = 0.011). Without distraction, the participants walked a further distance than with distraction (mean: 43.5 m vs. 40 m). According to the mean walking length, in both with distraction and without distraction the performance of the participants increased from the first to the second experiment day when using the road-crossing mode (with distraction first day: 31.25 m, second day: 37.5 m; without distraction first day: 41.25 m, second day: 46.25 m).

In the case of the angular rotation task, a three-way 3 × 2 × 2 RM-ANOVA with the dependent factors turning degree (90°/180°/360°), belt (without belt/compass mode) and day (first experiment day/second experiment day) was used. No significant main effects for day (*p* = 0.076), belt (*p* = 0.550) or turning degree (*p* = 0.187) and no significant interactions (*p* > 0.05) were present. When wearing the belt in compass mode, the participants remained below a turning error of 22.5° on the second day as on the first day (mean first day: 19.67°, second day: 15.29°), thereby indicating only a few mistakes in activating the correct vibromotor.

For the triangle completion task, a three-way 2 × 2 × 2 RM-ANOVA with the dependent factors belt (without belt/compass mode), distraction (without distraction/with distraction) and day (first experiment day/second experiment day) was calculated. No significant main effect for the factor day (*p* = 0.230), belt (*p* = 0.053) or distraction (*p* = 0.881) was found. There was, however, a significant interaction belt*distraction (*p* = 0.041). No further interactions of the factors were significant (*p* > 0.05). To examine the significant interaction more closely, two post hoc one-way RM-ANOVAs (without belt with distraction vs. without belt without distraction; with belt with distraction vs. with belt without distraction) were calculated by averaging over the experiment days. No significant difference could be found on comparing the distraction conditions without belt (*p* = 0.240), but a highly significant difference could be observed when comparing the distraction conditions with the belt in compass mode (*p* = 0.009). Since the mean of the absolute turning error of the condition with belt with distraction (mean: 33.75°) is lower than the mean without distraction (mean: 38.88°), the participants were significantly more precise in their angular turns with distraction than without.

**Figure 11:**
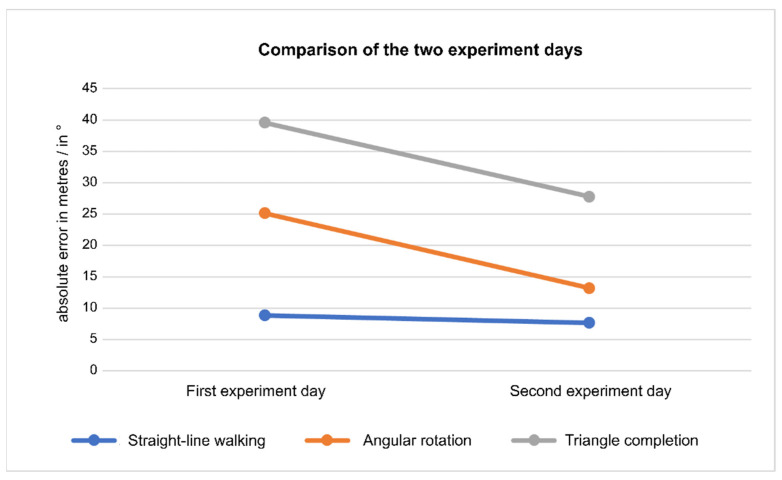
The mean absolute errors of the three experimental tasks straight-line walking (blue), angular rotation (orange) and triangle completion (grey) on the first and on the second experiment day are depicted. In the case of the straight-line walking task, the absolute error is the distance missing to the finishing line (in m). For the angular rotation task and the triangle completion task the absolute error is the absolute turning error (in °).

In sum, no significant difference between the two experiment days could be found in the individual analysis of the three experimental tasks. Jointly visualizing the error in three tasks reveals that the performance of the participants improved on the second experimental day (Figure 11). However, a priori we did not plan a statistical comparison of such pooled data. Thus, the indication that extended training with the belt might indeed have an effect on performance in the behavioural tasks has to await further targeted experiments.

## 4. Discussion

In this study, we evaluated the impact of the feelSpace belt on the emotional benefit in situations of traffic and the subjective experience of orientational abilities of the blind. We investigated the subjective sense of safety as well as the subjective reports of navigation abilities in outdoor situations with questionnaires. In the participants’ subjective evaluations, we found a significant decrease in discomfort ratings and a significant increase in confidence ratings in challenging situations of traffic or open spaces when participants were equipped with the belt. This suggests a crucial alleviation of stress associated with orientation and navigation for the blind. Additionally, behavioural experiments were conducted. We found that the concept of tactile compass signals is intuitively understood and that the benefits of the belt do not deteriorate through an additional distraction task. Importantly, our results show that the participants were objectively well adapted to orientation without visual cues, yet their subjective valuation indicated that the continuous feedback about their heading and their body turns from the feelSpace belt still had a large impact on feeling confident en route. Thus, we conclude that the belt is a promising assistive device for the blind and the visually impaired.

Emotionally handling the stress associated with orientation and navigation outdoors is a challenging situation for blind or visually impaired people. Crudden et al. [31] found in their study that 60% of the surveyed participants avoid entertainment and leisure activities and 50% limit their visits to family and friends due to the stress associated with reaching their destination. About one fourth of the participants were even limited in their partaking in working life. An aiding device that supports spatial navigation emotionally will thus have a large impact on independent participation in social life and quality of life of blind people [38]. In line with this point, participants reported an increased and lasting feeling of security when wearing the feelSpace belt that was already developed after one week. Most notably, in challenging situations like crossing a junction or an open space, or walking along a train platform, the belt significantly reduced the feeling of discomfort and increased participants’ confidence. We assume that the constant north signal of the belt enables also blind participants to get feedback on their body orientation and rotation and the direction of their movement during outdoor navigation and thus leads to the feeling of increased confidence and security. Previous research that investigated a teleguidance-based smart cane [16] or vibration information around the waist [35,39] also reported a positive emotional impact on blind participants. At the same time, participants do not have to handle the device actively, they just experience the continuous vibration as intuitive feedback about their body rotations. Further, participants reported an enhancement in their orientation and navigation abilities due to the belt. The positive ratings staying high throughout the study indicated that the belt was experienced as helpful over this period of time. Thus, in the presented study, participants developed a strong feeling that the belt’s information is helpful to master difficult orientation and navigation problems in everyday life reducing emotional stress in challenging outdoor situations.

Behaviourally, we conducted the straight-line walking task. This was designed to mimic the crossing of a large street or a square as especially challenging situations for the blind [12]. Our results revealed a better performance with the tactile belt in compass mode, which provides constant information of the north cardinal direction, in comparison to the road-crossing mode. This result suggests an intuitive understanding of the compass mode. In line with previous research [40], to benefit from the road-crossing mode participants have to go through training and learn how to adjust their bearing towards the goal in the environment. On comparing the two experiment days, results showed that the participants walked a significantly further distance without a distraction task than with distraction. Straight-line walking, although being an everyday task, thus profits from attentional resources of the blind participants despite of whether they are walking with a belt signal or not.

The angular rotation task tested the participants’ precision at making egocentric angular turns, their orientation abilities and their comprehension of the belt’s signal. Estimating bodily turns is important when circumventing an obstacle and reorienting to the original route. Already on the first experiment day, with and without the belt, turning errors mostly remained under 22.5°. These results indicate that the concept of the compass mode was intuitively comprehensible for the participants since a turning error of less than 22.5° indicates that the appropriate vibromotor was activated. Without the belt, this indicates that participants had already formed strategies (e.g., by positioning their feet in a perpendicular fashion while making the turn) prior to wearing the belt. As the belt’s precision level is 22.5° with 16 vibrating elements equally spaced around the waist, further improvement through the belt information would require mechanisms like hyperacuity and was not expected after the available training time. The feelSpace belt used in prior studies [35] was often equipped with 30 vibromotors, resulting in a leeway of 12°. There is, however, a trade-off between an improved accuracy of the belt and a higher cognitive demand of integrating the information when increasing the number of vibromotors [41]. Future studies may investigate whether equipping the belt with more vibromotors, with each vibromotor covering a smaller angle and thus inducing a higher cognitive load, would lead to more precise turns and an improvement for everyday use of blind participants.

As a third behavioural task, the participants performed a classical triangle completion task [5,6] which examined their navigation abilities in path integration and homing back to the start location. In contrast to previous research with sighted [32] and blind participants [35,36], our results revealed no significant performance improvements with the belt on the first experiment day. On the first experiment day, overall high estimation errors, independent of the condition, indicated that the task was difficult for the participants. Interestingly, when examining the two experiment days, the participants were significantly more precise with than without distraction when using the belt signal in compass mode. A possible explanation for this result might be that being distracted, they had to rely on the subconsciously integrated information of the belt [32], which led to more precise path integration than consciously memorising all sensory changes during the task. Thus, our results support the suggestion that the information of the belt can be used subconsciously in the triangle completion task with simultaneous distraction.

The behavioural part of the current study suffered due to the Covid 19 pandemic. As the behavioural investigations required travelling to the experimental location and meeting with the experimenters, only four of the late blind subjects were willing to participate in these. With this small number, a statistical evaluation using RM-ANOVAs is problematic. Nevertheless, we supply all analyses and take care to cautiously interpret the results. To improve on these points, a further investigation replicating our well-designed tasks with an increased number of participants should be performed once the pandemic is firmly under control. Still, in all three experimental tasks, a clear trend of an improved performance from the first experiment day to the second experiment day could be observed. Yet, for stronger claims regarding the improvement of orientational and navigational abilities through training with the belt further research with more participants needs to be conducted.

Tactile signals of the feelSpace belt have been investigated with sighted and blind participants (e.g., [33,34,35]). These studies showed that the belt induced changes in space perception and was beneficial in navigation and orientation tasks. Kärcher et al. [35] evaluated the tactile belt in a case study with a late blind subject. As with sighted participants, the signal of the belt gave additional helpful information during spatial navigation and was partly used without conscious attention. A further case study by Schumann [36] with one congenitally blind participant showed that new spatial relationships can be learned with the help of the belt in familiar environments. In sighted [33,34] and blind participants [35,36] the feelSpace belt was reported to especially bring about a high subjective feeling of safety during navigation in unknown as well as known outdoor environments. Consistently, many participants in the current study, triggered through the increased feeling of safety, also described situations in which they explored new routes and surroundings with the belt. Thus, the belt’s information animates and enables the blind participants to explore unfamiliar ground, expanding the range of independent everyday life activities.

Tactile signals are also used in sensory substitution devices which substitute the missing modality through another modality, e.g., vision through tactile information. The pioneer work of Bach-Y-Rita and colleges [19] revealed that blind participants could achieve to recognize and localize objects in the environment when actively handling a tactile sensory substitution device after a relatively short time of training. Since then, these findings could be supported also using other substituting modalities [20,21,22,23,24]. Even though the brain was shown to be plastic enough also in adult humans [25,42], sensory substitution devices are still not used frequently by blind people [26,27]. The feelSpace belt, starting out as a tool to investigate sensory augmentation [32], does not fulfil the criteria of a sensory substitution device as it does not aim to replace the lost visual capacity. Rather, the tactile belt, giving information about magnetic north, supplies information about the environment that helps blind people to adapt to wayfinding without vision. The information about relative heading and body turns received through the belt is not achieved through other aiding devices blind people commonly use.

The most important tool for outdoor navigation is the cane. The cane scans the immediate environment for obstacles and orientation cues, like the curb stone or tactile tiles on the floor [9]. There are further developments of the cane [17], e.g., the EyeCane that enables the detection of obstacles in different heights and distances [27] or the WeWalk smart cane that gives feedback about distant obstacles via ultrasonic sensors and acoustic signals [18]. Yet the cane cannot give continuous information about cardinal heading and both detection of and correction for involuntary body turns are effortful with the cane alone [10]. Smartphone Apps or speaking compasses can inform about the current heading but are only used at intervals in everyday application. While a continuous use may be imaginable, it would constantly block the important auditory sense with an information of comparatively low urgency [11]. The feelSpace belt provides constant cardinal information, keeping hands and ears free so that, e.g., communication or hearing important signals in the environment are unaffected. Thus, the continuous monitoring of current cardinal heading through vibrotactile signalling of the feelSpace belt constitutes an addition to commonly used navigation devices to support the blind in everyday challenges. This approach is characterised by a pronounced emotional benefit which encourages mobility.

In the context of this research on its emotional impact, the feelSpace belt was used as a tactile compass. As an everyday aiding device, it cannot only give compass directions for intuitive feedback about body rotations but can also be used as a navigation device, signalling turn-by-turn navigation information. Many Orientation and Mobility instructors in Germany already use the tactile feedback on body rotation to help their blind clients to adapt to life without vision.

This study shows the value of incorporating both first-person qualitative data and quantitative data of behavioural tasks by proposing an extended version of a successfully tested experimental set-up [35] and shaping it more to cover everyday experiences of the participants. This set-up permitted a holistic picture of the experience with the belt and the implementation into everyday behaviour.

## 5. Conclusions

Summing up, the present study exemplifies the potential of the feelSpace belt as an assistive device for the blind and visually impaired especially through the pronounced emotional benefit. Further strong points are the independence from attentional resources as well as keeping the hands and ears free while ensuring easy integration into existing orientation and navigation strategies. Considering the great advances of modern AI and machine learning, such assistive devices with outputs based on vibrotactile signalling could go beyond existing improvements, further enhancing mobility and thereby allowing for greater participation of blind and visually impaired people in all aspects of social life such as education, labour market and civic life.

## Data Availability

The data presented in this study are available on request from the corresponding author.
